# Paradoxical Thromboembolic Ischemic Stroke Following Tissue Plasminogen Activator Instillation for Clogged Central Venous Dialysis Catheter

**DOI:** 10.7759/cureus.20346

**Published:** 2021-12-11

**Authors:** Ifeanyi Nwosu, Emeka Ibeson, Sehajpreet Singh, Ranbir Singh, Amit Gulati, Dmitriy Zadushlivy, Yizhak Kupfer, Anna Derman, Britney Clemen, Arjun Basnet, Geraldine Nsofor, Annabel U Ogar

**Affiliations:** 1 Internal Medicine, Maimonides Medical Center, New York, USA; 2 Critical Care Medicine, Maimonides Medical Center, New York, USA; 3 Critical Care, Maimonides Medical Center, New York, USA; 4 Neuroradiology, Maimonides Medical Center, New York, USA; 5 Internal Medicine, Milton Keynes University Hospital, Milton Keynes, GBR; 6 Internal Medicine, Peterborough City Hospital, Peterborough, GBR

**Keywords:** ischemic stroke, paradoxical embolism, patent foramen ovalis, tissue plasminogen activator, central venous dialysis catheter

## Abstract

Central venous catheters including dialysis catheters are a potential source of venous thrombosis and pose a risk for paradoxical embolic events including ischemic stroke and systemic embolism in patients with a patent foramen ovale (PFO). The adult population with a PFO and patients with a central venous dialysis catheter (CVDC) are at increased risk of a paradoxical embolic event. Since bubble study is not routinely done during echocardiogram in a patient with CVDC, it is difficult to identify at-risk patients for paradoxical embolic events during catheter manipulation, especially for clogged CVDC.

We report a rare case of a 79-year-old lady with end-stage renal disease on hemodialysis (HD) using a CVDC who developed a paradoxical embolic ischemic stroke following the use of tissue plasminogen activator (tPA) for unclogging a dialysis catheter. We aimed to highlight the existing risks of thromboembolism associated with the long-term use of central CVDC, especially the potential risk of paradoxical embolism and ischemic stroke with the use of tPA for management of clogged dialysis catheters. We emphasize the questionable need for a bubble study echocardiogram in all patients requiring long-term dialysis catheters.

## Introduction

Paradoxical thromboembolism is a venous thrombosis causing systemic embolization through a right to left cardiac shunt; about 70% occur through a patent foramen ovale (PFO) [1,2]. PFO is the persistence of the normal fetal interatrial communication postnatally and represents a potential channel for paradoxical systemic arterial thromboembolism, such as ischemic stroke and other ischemic events [3]. The incidence of PFO in the adult population is about 25% [4,5]. Most cases are incidentally found following the evaluation of patients with cryptogenic stroke and this association is more common in young adults with ischemic stroke compared to older patients [6].

Although paradoxical embolism through a PFO is a common cause of ischemic stroke, paradoxical embolic stroke associated with a central venous dialysis catheter (CVDC) is relatively uncommon and only a few cases among adult patients have been reported in the literature [7-11]. To the best of our knowledge, this is the first case of paradoxical embolism associated with the use of a tissue plasminogen activator (tPA) for unclogging a hemodialysis (HD) catheter. Clogging of dialysis catheters is frequently encountered in clinical practice. Among numerous causes of catheter dysfunction in HD patients, thrombotic occlusion of catheter occurs in 30-40% at various stages from within 24 hours of catheter insertion and beyond [12]. Non-invasive management of a clogged dialysis catheter typically employs the use of fibrinolytic agents such as tissue plasminogen activator (tPA) like alteplase and has proven to be effective [13-15].

The current technique for unclogging of dialysis catheter using tPA involves the use of 2 ml of alteplase (1 mg/ml concentration) instilled into each catheter lumen, care taken not to push the solution into the systemic circulation as in the case of flushing venous access. The catheter is then aspirated about 30 minutes later to ensure easy aspirate from each catheter lumen [16]. Even with the best technique, there exists a possibility of embolization of a thrombus into the systemic venous circulation through the right side of the heart causing a pulmonary embolism, but in a patient with a PFO, there is an additional risk of paradoxical systemic arterial embolization.

## Case presentation

**Figure 1:**
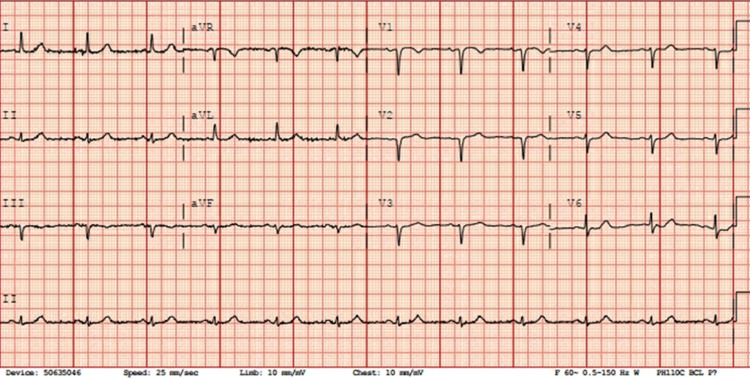
EKG showing sinus rhythm, old anterior Q waves, and poor R wave progression. aVR: augmented vector right; aVF: augmented vector foot; aVL: augmented vector left

A 79-year-old woman with a past medical history of Alzheimer’s dementia, type 2 diabetes mellitus, sarcoidosis on prednisolone, hypothyroidism, and end-stage renal disease (ESRD) on HD through a right internal jugular vein permcath, hypertension, and hyperlipidemia presented to the emergency department (ED) with a clogged dialysis catheter. She missed her last HD due to a catheter malfunction. She has no other symptoms or complaints and reported feeling well. The patient walked into the ED accompanied by a family member. The patient at her baseline is forgetful and occasionally confused but able to carry out most of the activities of daily living. Vital signs on admission were as follows: blood pressure (BP) 104/90, heart rate (HR) 66 beats per minute (BPM), respiratory rate (RR) 20 breaths per minute (BPM), temperature 98.8°F, SpO_2_ 95% on room air. The ED patient had alteplase instilled into the dialysis catheter, followed by a dramatic bout of coughing just after the infusion. She became unresponsive with a Glasgow Coma Scale score of 8 and was hypoxic with the lowest oxygen saturation of 70%. The patient was intubated for airway protection and ventilated. A 12-lead electrocardiogram (EKG) obtained immediately showed the patient was in sinus rhythm (Figure 1).

Urgent non-contrast CT head showed no acute infarction, intracranial hemorrhage, or mass lesion. CT angiogram chest was negative for pulmonary embolism. Findings of small bilateral pleural effusion, extensive calcified mediastinal, and bilateral hilar lymphadenopathy similar to previous CT images were noted. Extensive laboratory workup was done and pertinent laboratory findings are shown in Table 1.

**Table 1:** Pertinent laboratory results. HSV: herpes simplex virus; VZV: varicella-zoster virus; PCR: polymerase chain reaction; eGFR: estimated glomerular filtration rate; CSF: cerebrospinal fluid; VDRL: venereal disease research laboratory; LDL: low-density lipoprotein

Hematology/Biochemistry/Immunology	Result	Reference Range
Hemoglobin (g/dl)	8.6	12-16
White blood cells (K/ul)	4.3	4.8-10.8
Platelets (K/ul)	376	150-450
Sodium (mmol/l)	141	135-149
Potassium (mmol/l)	3.8	3.4-4.8
Urea (mg/dl)	31.1	7-21
Creatinine (mg/dl)	7.1	0.3-1.1
eGFR (ml/min/1.73 m^2^)	7	>60
Troponin I (ng/ml)	0.03, 0.04	0.00-0.04
LDL-cholesterol (mg/dl)	165	40-100
Hemoglobin A1C, plasma	6.5	(4-6%)
Ammonia (umol/l)	28	16-63
CSF glucose (mg/dl)	105	40-170
CSF protein (mg/dl)	52	20-100
HSV, VZV PCR	Negative	Negative
West Nile virus/CSF IgM	Negative	Negative
CSF VDRL	Non-reactive	Non-reactive
CSF cryptococcus antigen	Negative	Negative
Oligoclonal banding CSF and serum	Negative	Negative
Beta-2-glycoprotein-1 IgG, IgM, IgA	Negative	Negative
Serum immunoglobulin G level (mg/dl)	442	610-1660

**Figure 2:**
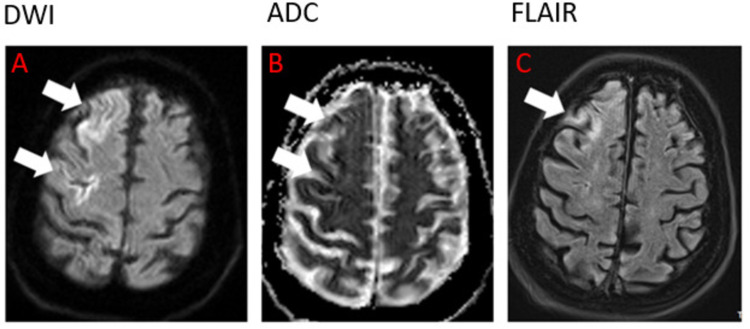
Brain MRI images (DWI, ADC, and FLAIR) showing an acute right frontal hemispheric stroke. DWI: diffusion-weighted imaging; ADC: apparent diffusion coefficient; FLAIR: fluid-attenuated inversion recovery

Neurology and stroke teams consulted and an MRI brain was ordered for exclusion of acute stroke. A lumbar puncture was done pending further evaluation for loss of consciousness. CSF studies are shown in Table 1 above. Electroencephalogram did not reveal any focal slowing, epileptiform potentials, or seizure activities. Initial echocardiogram without bubble study showed normal left ventricular systolic (LV) function with ejection fraction 61-65%, mild LV diastolic dysfunction, and compared to her previous studies, a mildly dilated right atrium and ventricle. Venous Doppler study of the lower extremities was negative for deep vein thrombosis. The patient was admitted to the medical intensive care unit for further management and evaluation. The patient was extubated satisfactorily 24 hours after admission and tolerated oxygen supplementation using a nasal cannula. Post-extubation, it became apparent that the patient had weakness of the left upper and lower extremities, with hypertonia and positive Babinski reflex. She was more confused compared to her baseline mental status. A repeat CT head, and a CT angiogram head, and neck were negative for acute infarction, intracranial hemorrhage, or mass lesion. MRI brain showed gyriform-restricted diffusion with fluid-attenuated inversion recovery (FLAIR) hyperintensity in the right frontal cortex most likely an acute infarct (Figures 2A-2C).

**Figure 3:**
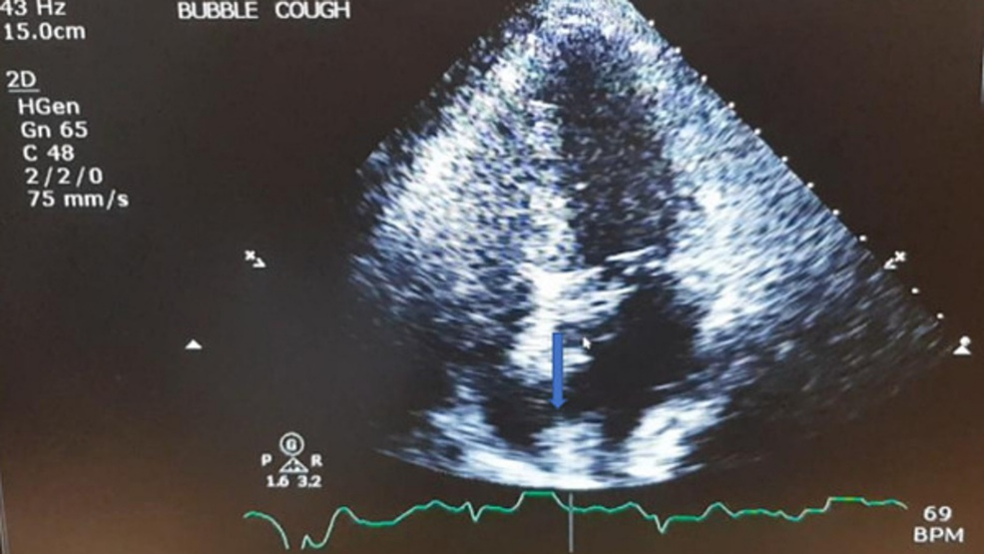
Bubble study echocardiogram showing a PFO. PFO: patent foramen ovale

She was diagnosed with right frontal hemispheric ischemic stroke with left hemiparesis and commenced on aspirin and high-intensity statin. With the high suspicion for paradoxical embolic stroke entertained, a repeat echocardiogram with bubble study was ordered and cough provocation showed evidence of a right to left shunt (frame 105/117) consistent with a moderate PFO (Figure 3). The patient continued further post-stroke care and was transferred to the medical floor for further management.

## Discussion

Under the physiologic condition, left pressures are greater than the right, and PFO show left to right shunt. This pressure gradient can be transiently reversed in certain conditions that increase the right atrial pressure such as Valsalva maneuver, coughing, squatting, and defecation, which causes a right to left shunt that can lead to paradoxical embolism into the systemic circulation causing an ischemic stroke or other arterial embolisms. In our patient, the cause of a right to left shunt most likely was acute but could be chronic. She also has a diagnosis of sarcoidosis in the past that can lead to chronic pulmonary hypertension and increased right heart pressures causing a chronic reversal of the shunt. After tPA was instilled, she had bouts of cough which can cause an acute reversal of shunt from right to left. Also, she might have had a pulmonary embolism of which cough is a symptom and can lead to an acute reversal of shunt. The calculated risk of paradoxical embolic stroke (RoPE) score for the patient was low (2), suggesting a 0% chance that the stroke is due to PFO, and a 20% risk of a two-year recurrence of stroke/transient ischemic attack. The uniqueness of this case compared to the few reported cases of paradoxical embolic stroke associated with central venous catheter thrombosis is the relationship between the use of tPA for unclogging catheters and the apparent physiologic effect of coughing or maneuver that may affect right heart pressures [7,8,11].

Stroke risk in patients with PFO is a continuum as with our patient. The recommended management of patients with PFO and cryptogenic stroke is the closure of PFO in patients of 60 years and below [4,17]. No consensus guideline exists for the closure of PFO in older patients and the use of long-term prophylactic anticoagulation is controversial. Bleeding risk remains a concern for the long-term anticoagulation in patients with ESRD on HD and careful consideration must be given before commencement of anticoagulation based on the risk and benefits especially the risk of major bleeding versus recurrent ischemic stroke. Our patient continued antiplatelet therapy and the use of a tPA locking solution to prevent catheter dysfunction.

## Conclusions

Paradoxical embolic events including embolic stroke through a PFO remain a concern in ESRD patients with long-term dialysis catheters, especially in association with catheter dysfunction and its management. No study has documented the incidence of PFO among patients with ESRD, and as a result, it may be reasonable to consider the creation of guidelines or recommendations for the use of tPA in patients with CVDC, like patient education including proper breathing techniques to prevent an increase in pulmonary pressure. We are not sure a routine screening bubble study echocardiogram in all patients with a CVDC is cost-effective. We could consider bubble study echocardiogram in patients suspected to have increased pulmonary pressure or PFO before these procedures. But if we found a PFO with the reversed flow, what are we to do with a clogged CVDC? Caution is therefore advised when using tPA for unclogging catheters, and in general, educating patients on avoiding maneuvers that may increase right heart pressures may help minimize the risk of paradoxical embolic stroke.
